# Plastics and the Sustainable Development Goals: From waste to wealth with microbial recycling and upcycling

**DOI:** 10.1111/1751-7915.14459

**Published:** 2024-04-08

**Authors:** Marco A. Pereyra‐Camacho, Isabel Pardo

**Affiliations:** ^1^ Centro de Investigaciones Biológicas Margarita Salas, CSIC Madrid Spain; ^2^ Interdisciplinary Platform SusPlast, CSIC Madrid Spain

## Abstract

Plastics pollution has become one of the greatest concerns of the 21st century. To date, around 10 billion tons of plastics have been produced almost exclusively from non‐renewable sources, and of these, <10% have been recycled. The majority of discarded plastic waste (>70%) is accumulating in landfills or the environment, causing severe impacts to natural ecosystems and human health. Considering how plastics are present in every aspect of our daily lives, it is evident that a transition towards a Circular Economy of plastics is essential to achieve several of the Sustainable Development Goals. In this editorial, we highlight how microbial biotechnology can contribute to this shift, with a special focus on the biological recycling of conventional plastics and the upcycling of plastic‐waste feedstocks into new value‐added products. Although important hurdles will need to be overcome in this endeavour, recent success stories highlight how interdisciplinary approaches can bring us closer to a bio‐based economy for the sustainable management of plastics.

The way we produce and consume plastics today is a prime example of how decades of unsustainable practices have caused profound and possibly irreversible changes to our environment. Since the start of the large‐scale commercialization of plastics in the 1950s, annual production has increased by roughly 200‐fold (Geyer et al., 2017), reaching 400.3 million tons in 2022. Of these, 90% accounted for virgin material that was newly synthesized from fossil feedstocks (Plastics – the fast Facts, 2023). According to some estimates, the share of global oil consumption dedicated to the production of plastics will reach 20% by 2050 (Ellen MacArthur Foundation, 2017). Our dependency of plastics is explained by their versatility, strength, durability, and cost‐effectiveness, making them ideal materials for a myriad of applications. However, the irresponsible use of plastics – in particular single‐use plastics – comes with dire costs to the environment and human health. Current plastic recycling rates worldwide are around 10%, while approximately 18% is incinerated. The rest is discarded in landfills (50%) or littered into the environment (22%), where they break down into micro and nanoplastics that can adsorb toxic chemicals and pathogens, leach additives with endocrine disruption properties, be ingested by fauna, and eventually enter the food chain, among other hazards (Jiménez‐Arroyo et al., 2023). It is estimated that ~10 million tons of plastic reach our oceans every year, and its recovery seems impracticable (MacLeod et al., 2021; Stubbins et al., 2021).

The concern caused by the mismanagement of plastics is evidenced by the historical UN resolution in March 2022 to elaborate an international legally binding agreement to end plastic pollution (UNEA Resolution 5/14). In contrast, it is quite surprising that only one indicator of the 17 SDGs (14.1.1b) makes an explicit mention to plastic debris, in the context of target 14.1 to “prevent and significantly reduce marine pollution of all kinds, in particular from land‐based activities, including marine debris and nutrient pollution”. Nonetheless, rethinking how we produce, consume, and manage plastic waste can make important contributions to achieving several of the SDG targets, if we consider their global scope. From an environmental perspective, preventing and dealing with uncontrolled leakage of plastic waste directly affects SDGs 6 (clean water and sanitation), 14 (life below water), and 15 (life on land), whereas the production of more easily recyclable or biodegradable materials from renewable sources relates to SDGs 9 (industry, innovation and infrastructure), 11 (sustainable cities and communities), 12 (responsible consumption and production), and 13 (climate action). Undoubtedly, microbes can offer solutions to mitigate plastic pollution and aid in the transition from a linear to a circular economy of plastics in numerous ways, as summarized in Table 1. Nevertheless, here we will focus on the use of microorganisms and their enzymes as an alternative way to recycle and bring value to today's plastic waste.

**TABLE 1:** Microbial biotechnology contributions to the SDGs in the context of plastics.

SDG	Problems caused by plastics	Opportunities for microbial biotechnology
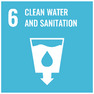	Presence of micro‐ and nanoplastics in drinking water Leaching of toxic additives (e.g. phthalates) Microplastics as carriers of pathogens	Biodegradation of microplastics and additives in water treatment facilities Low‐cost biosensors for contaminants
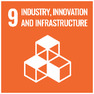	Dependency on fossil feedstocks Energy‐intensive production processes	Bioplastic production from renewable, syngas, CO_2_, and municipal waste feedstocks
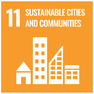	Excessive generation of plastic waste Accumulation of plastic waste in landfills	Biological recycling of conventional plastics New bioplastics that are chemically recyclable by design Compostable and biodegradable plastics Biological fixation of CO_2_ to produce bioplastics
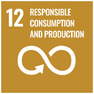	Single‐use plastics with short useful lifespan Low recycling rates Linear life cycle	
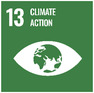	CO_2_ generation during extraction, production, and incineration of plastic waste	
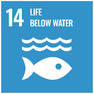	Littering and accumulation of plastics in oceans and soils Ingestion by fauna Generation of micro‐ and nanoplastics Perturbation of native microbial communities	Controlled biodegradation in case of accidental spillage Biocompatible bioplastics Non‐hazardous, bio‐based additives
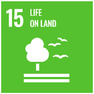		

Plastics' stability and resistance to degradation are advantageous features that account for their widespread use. However, these same traits become an issue when the time plastic waste persists in landfills (or worse – the environment) is tens to hundreds of times longer than their useful lifetime (Chamas et al., 2020). To date, mechanical recycling is the most cost‐effective technology to re‐introduce plastic products into the value chain, although its limitations are well known (Uekert et al., 2023). Primarily, the loss of material properties during each re‐processing cycle and the inability to deal with composites and mixed polymer streams. These shortcomings have motivated numerous efforts to develop alternative technologies that can complement mechanical recycling. Among them, chemical or tertiary recycling using hydrolytic enzymes to recover polymerizable monomers emerges as an enormous opportunity for microbial biotechnology, which has the advantage of being less energy intensive and generating less undesired side‐products than other chemical recycling processes (e.g. pyrolysis and solvolysis).

Enzymatic recycling of conventional plastics has mainly been circumscribed to the polyester polyethylene terephthalate (PET), which is largely used in textiles, beverage bottles, and food packaging. This approach allows the recovery of its constituent monomers terephthalic acid and ethylene glycol, which can be readily repolymerized to synthesize recycled PET with virgin‐like properties. Hundreds of scientific papers on the use of microbial PET hydrolytic enzymes have been published since the early 2000s (Kim et al., 2022), and large‐scale enzymatic recycling of PET is closer to becoming a reality with the expected inauguration of a commercial plant in 2025 capable of treating 50,000 tons of post‐consumer PET per year (https://www.carbios.com/en/carbios‐to‐build‐in‐france‐its‐plant/, accessed 13 February, 2024). This milestone has been possible thanks to roughly 20 years of interdisciplinary research that spans bioprospection, protein engineering, computational biology, process engineering, techno‐economic analysis, and life cycle assessment. Furthermore, it has prompted an intense search for new enzymes that are active on other widely used plastics (Zhu et al., 2022). As an example, “urethanases” and “nylonases”, which are active on oligomers of polyurethanes (8% of plastics) and polyamides (5.8% of plastics), could open the door to the enzymatic recycling of these polymers (Bell et al., 2024; Branson et al., 2023; Negoro et al., 2021; Puetz et al., 2023). Although this possibility is still in a preliminary stage, the lessons learned from PET hydrolases will surely accelerate the progression from laboratory settings to industrial facilities for the enzymatic recycling of a growing number of plastics and textiles.

Of course, enzymatic degradation of the aforementioned plastics is facilitated by the presence of hydrolyzable linkages. By contrast, the enzymatic depolymerization of plastics with a C–C backbone poses a substantial challenge due to the inert character of this chemical bond. This is the case for polyethylene (PE), polypropylene (PP), polystyrene (PS), and polyvinylchloride (PVC). It is important to highlight that polyolefins alone (PE and PP) account for 46% of global plastics production and are the base polymers for 69% of packaging plastics, making them the main contributors to plastics pollution. Among these polymers, biodegradation of PE has been studied more extensively. It is generally accepted that degradation of PE in the environment starts with abiotic weathering and deterioration mediated by mechanical erosion, temperature, UV light, and oxidation, which randomly introduce carbonyl groups in the polymer chain (Chamas et al., 2020). This fact has led some authors to propose oxygenases, peroxidases, peroxygenases, and laccases as biocatalysts to introduce oxygen atoms in the polymer chain. The concerted action of these enzymes together with low molecular mass radicals and ions acting as mediators could lead to a biodegradation process analogous to that of lignin from plant biomass – a highly recalcitrant natural polymer (Chen et al., 2020). Unfortunately, the numerous attempts to use isolated microbial enzymes capable of degrading pristine plastics (mainly PE and PS) have mostly been unsuccessful thus far, and abiotic pre‐treatment of the substrate is usually required to detect substantial biodegradation (Zhang et al., 2022). Additionally, the numerous additives used during plastics manufacturing have raised concerns on whether there is true enzymatic depolymerization and to what extent these substances affect microbial degradation. Regardless, the oxidative depolymerization of PE does not release discrete monomers that can be directly used to re‐synthesize the original polymer (i.e. ethylene), and the diversity and mixture of possible degradation products (alkanes, ketones, alcohols, and carboxylic acids of varying chain length) also poses a technical challenge when evaluating the biodegradation of these plastics. Indeed, most of the published works related to the microbial biodegradation of PE rely on indirect methods such as microscopic, gravimetric, and infrared spectroscopy analyses to evaluate biofilm formation and changes to the mass and surface of the plastic. However, these changes could be caused by unspecific deterioration mediated by a number of molecules released from microbial metabolism (e.g. organic acids, reactive oxygen species) (Zadjelovic et al., 2022). In the search for PE depolymerizing enzymes, standardized additive‐free substrates and analytical methods that allow the detection and identification of degradation products will need to be implemented within the research community to accurately discriminate between true depolymerization and the transformation of other components present in plastics.

In any case, fast and full depolymerization of untreated plastic waste by microbes or their enzymes alone is highly unlikely, even when using engineered enzymes under favourable conditions (Chow et al., 2022). Enzymatic attack is constrained by the inherent properties of the plastic material, i.e. surface area, hydrophobicity, and crystallinity. Thus, in the context of enzymatic recycling of plastics, mechanical, thermal, or chemical pre‐treatments will be required to achieve complete depolymerization in a time‐effective manner, which can in turn raise process costs. However, this drawback could be overcome with the biological conversion or upcycling of plastic waste into value‐added products, another major opportunity for microbial biotechnology (Tiso et al., 2022). Indeed, the strategy of combining thermochemical and biological processes for the deconstruction of recalcitrant polymers and the production of valuable compounds has been pursued for decades in the context of lignocellulosic biomass valorization (Weiland et al., 2022).

To date, most works on the biological upcycling of plastics have focused on the microbial conversion of pre‐treated monolithic polymers – mainly PET and, to a lesser extent, PS and PE. The processes employed for plastics deconstruction include pyrolysis, glycolysis, alkaline hydrolysis, microwave‐assisted, and enzymatic depolymerization (for some examples on PET, see Bao et al., 2023; Kenny et al., 2008; Kim et al., 2019; Tiso et al., 2021; Werner et al., 2021). More recently, oxidative thermochemical depolymerization of mixed PET, PE, and PS has been used to generate a mixture of organic acids (TPA, aliphatic dicarboxylic acids, and benzoic acid) that were converted by an engineered bacterial strain into a single product, following a “biological funnelling” approach (Sullivan et al., 2022). This strategy takes advantage of the metabolic versatility of microbes to convert a complex mixture of substrates into a single product of interest. Although still a proof‐of‐concept demonstration, the great potential of this approach is that it is theoretically compatible with any chemical recycling technology that deconstructs mixed plastic polymers into low molecular mass compounds that can be assimilated by microorganisms, such as pyrolysis. Pyrolysis is a mature chemical recycling technology based on the depolymerization of plastics at high temperature and in the absence of oxygen, generating gas, pyrolysis oils, and solid char residues. The product profile obtained from pyrolysis is highly dependent on feedstock composition and is quite sensitive to contaminants, so most current commercial processes are centered around recycling PE and PP. Pyrolysis of these plastics mostly generates medium and short‐chain length olefins, which could be assimilated by alkane‐utilizing bacteria that have been studied for many years as bioremediation agents for oil spillages. Nonetheless, one of the major challenges for the biological valorization of pyrolysis oils is the low solubility in aqueous media and its acute toxicity to microorganisms (Arnold et al., 2017).

Many of the aforementioned examples use monocultures of non‐modified or engineered bacteria to carry out the conversion of depolymerized plastics. However, the use of microbial consortia to individually transform each depolymerization product might be beneficial to the bioconversion process by reducing the metabolic burden on a single strain and increasing tolerance to high substrate loadings (Bao et al., 2023). Although this option holds great potential, a deep understanding of microbial interactions and population dynamics will be needed to establish robust and stable processes, especially when using synthetic consortia. Regardless, by combining thermochemical deconstruction with bioconversion, the economic viability of alternative plastic recycling processes could be improved and serve as an incentive to treat post‐consumer waste of lower quality that is not adequate for mechanical recycling. Additionally, they could help to significantly reduce the accumulation of plastic waste, since they could enable the recycling of composite and multipolymer materials that cannot be easily sorted and recycled with current technologies and thus usually end up landfilled or incinerated.

Biological conversion is also an attractive solution to an often‐overlooked limitation to the mechanical recycling of plastics: additives. Hundreds of substances of different chemical nature are added during the manufacturing of plastics to impart different properties that improve processability and performance: UV and heat stabilizers, antioxidants, antistatic agents, flame retardants, plasticizers, lubricants, etc. Unfortunately, the precise formulation of the majority of plastic products is obscure, and some of the most historically used additives are now considered substances of concern (Wiesinger et al., 2021). This is the case for some of the phthalate esters most widely used as plasticizers during the production of polyvinyl chloride (PVC). In particular, the use of di‐2‐ethylhexyl phthalate (DEHP), diisononyl phthalate (DINP), di‐n‐butyl phthalate (DBP), and diethyl phthalate (DEP) has been banned in several nations since they are known endocrine disruptors and toxic for reproduction. Thus, PVC plastics containing these legacy plasticizers cannot be recycled unless they are removed. Additionally, land‐filling is not a safe option since phthalates – which are not covalently bound to the polymer – can leach out and contaminate waters and soils. Solvent‐based extraction is a possible technology to remove plasticizers and recover clean PVC polymer (Ügdüler et al., 2020). The phthalate‐enriched streams obtained could potentially be assimilated by wild‐type or engineered bacteria harbouring phthalate degradation pathways (Boll et al., 2020; Pereyra‐Camacho et al., 2021). As discussed for chemical recycling, microbial upcycling of plasticizers could serve as an incentive to recycle PVC using this technology. Indeed, the first PVC recycling plant based on the VinyLoop dissolution process closed in 2018 after the implementation of legislation from the European Union banning the use of hazardous phthalates in PVC products, 16 years after its inauguration. One of the reasons for the closure was that the recovery of legacy plasticizers for their elimination was not economically viable.

Having argued the interest of using plastic waste as feedstock in microbial processes, the question then is what to produce. In pursuit of a circular bioeconomy of plastics, the production of bioplastics from conventional plastic waste is an obvious choice. The different types of bioplastics and their end‐of‐life management options are discussed in more detail by Serrano‐Aguirre and Prieto (2024) in an accompanying editorial. Particularly, the above‐mentioned plastic upcycling works have targeted the production of biopolymers naturally synthesized by bacteria (i.e. polyhydroxyalkanoates) or different monomers that can be used as precursors for catalytic polymerization, e.g. muconic acid, β‐ketoadipic acid, and hydroxyalkanoyloxy‐alkanoates. Nevertheless, it is quite straightforward to envision the production of many other polymerizable monomers from plastic‐derived feedstocks by introducing appropriate catabolic and biosynthetic pathways to convert these alternative carbon sources into different metabolic intermediates (Chae et al., 2020; Johnson et al., 2019). For example, lactate, succinate, and 1,4‐butanediol for the production of biodegradable polymers that are currently in the market, i.e. polylactic acid (PLA), polybutylene succinate (PBS), polybutylene succinate‐*co*‐adipate (PBSA), or polybutylene adipate‐*co*‐terephthalate (PBAT). For many years, substantial efforts have been dedicated to implement the large‐scale biological production of these compounds through metabolic and process engineering approaches using simple substrates (e.g. glucose), although 1,4‐butanediol and succinate are still largely sourced from fossil feedstocks. On the other hand, completely new bio‐based polymers that have other advantageous properties are also actively being developed, such as PET‐like polymers based on furan or pyridine dicarboxylic acids (Pellis et al., 2019), nylon‐like polyamides based on aliphatic dicarboxylic acids and diamines (Lee et al., 2023; Rorrer et al., 2022), and PE‐like polyesters and polycarbonates based on long‐chain fatty acids (Häußler et al., 2021), to name a few.

In all, the concept of using microbes to convert recalcitrant plastic from non‐renewable feedstocks into recyclable or biodegradable bioplastics is especially appealing in the context of sustainable development. Unfortunately, so far, it has been extremely difficult to introduce new bioplastics to the market. The reality is that, today, producing plastics or other bulk chemicals by biological means has low profit margins at best, and petroleum‐based plastics are particularly cheap to make. Indeed, it is improbable that bio‐based materials will be able to satisfy the ever‐growing global demand for plastics, or that a bio‐based/biodegradable alternative will be available for every application. Although several policies and consumer demands are pushing the market to increase the use of bio‐based and recycled plastics, in the near future it may be more economically viable to use biology to convert plastic waste into specialty chemicals with smaller market volumes and higher selling price, such as surfactants, food additives, etc. In this way, foundational technologies and infrastructures could be put in place to demonstrate the scalability and viability of the biological upcycling of plastics.

## CONCLUDING REMARKS

The global challenges we face due to the misuse and mishandling of plastics are multi‐faceted and enormous in scale. As such, there is no silver bullet to deal with the threat posed by plastics pollution. Unfortunately, it is unlikely that we will ever be able to recover the plastic debris that have already spread into all of Earth's ecosystems and environments, and in the future, we will still depend on these materials in almost every aspect of our daily lives. In view of this conundrum, it is urgently critical that we transition from a linear to a circular paradigm for plastics to avoid further harm, and increasing concern among consumers, producers, and policymakers is pushing in this direction. Current recycling strategies lack a multidisciplinary approach to effectively manage the complexity of plastic waste streams at the end of their lifecycle, meaning that only a small fraction returns to the value chain. It is thus necessary to focus efforts on the development of alternative recycling technologies and the design of materials with a closed‐loop life cycle. In this endeavour, the progress made during the last decades around the enzymatic‐assisted deconstruction of recalcitrant polymers (plastics and lignocellulose), the biological funnelling and conversion of complex feedstocks, and the metabolic engineering for improved production of compounds of interest will be instrumental to offer viable alternatives to the current economic model of plastics. Certainly, microbial biotechnology has made and will continue to make essential contributions to the sustainability of plastics, although it will need to advance hand in hand with complementary disciplines to deliver additional real‐world solutions outside of academic research. This holistic view will be key to redefine how we produce and consume plastics in the future.

## AUTHOR CONTRIBUTIONS

**Marco A. Pereyra‐Camacho:** Writing – original draft; writing – review and editing. **Isabel Pardo:** Conceptualization; funding acquisition; writing – original draft; writing – review and editing.

## CONFLICT OF INTEREST STATEMENT

I.P. is co‐inventor of patent applications US20210285019 and US20220089654, which describe the use of engineered bacteria for the conversion of PET.
